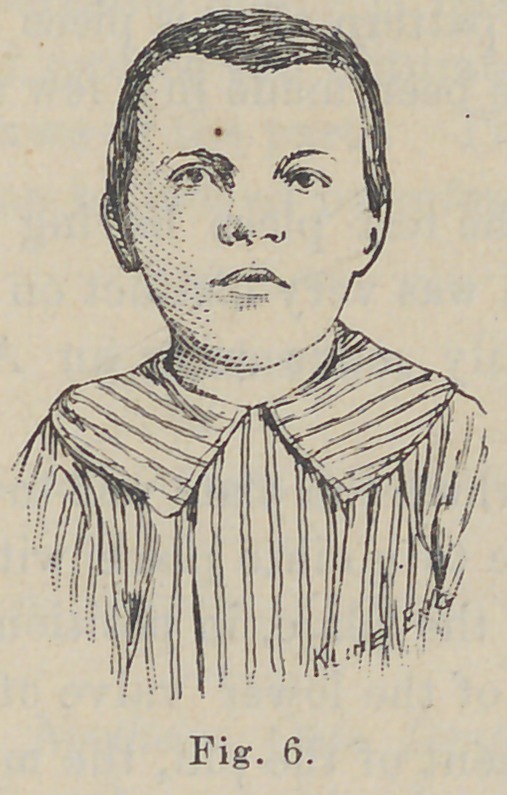# Treatment of the Congenital Deformity of Cleft Palate

**Published:** 1893-06

**Authors:** Weston A. Price

**Affiliations:** U. of. M., Ann Arbor, Mich.


### THE DENTAL REGISTER.

VOL. XLVII.] JUNE, 1893. [No. 6.

### COMMUNICATIONS.

### Treatment of the Congenital Deformity of Cleft Palate.

BY WESTON A. PRICE, U. OF. M., ANN ARBOR, MICH.

No affliction to which man is heir is more humiliating than that of congenital deformity, and of these none is more mortifying than that of hare-lip and cleft palate. Almost before these individuals come to appreciate that they are different from their fellows, they have learned to hide their faces that they may not see the look of disgust with which they are invariably met. Since this deformity can not be hid it must be corrected. Unfortunately this branch of surgery has not received an adequate amount of attention, not, however, because it is considered of little importance, but because of the complications and difficulties in the way of operating. It is practicable to render great service to persons so afflicted, either by the adaptation of an appliance or an operation, or both.

The following is a description of a case treated at the University of Michigan during the last session:

Willie Snyder, aged seven years, came to Dr. H. L. Obetz, of the Homeopathic Department, for treatment. The case presented a congenital deformity of cleft-palate and hare-lip. The cleft extended entirely through the hard and soft palate, making the oral cavity continuous with that of the nares. From the back, and forward, the course was in the median line to the pre-maxillary bones and thence it followed the left pre-maxillary suture, appearing on the face at the left nostril. Unfortunately no picture was taken showing the external appearance before the operations were begun. The alveolar process of the right border of the cleft extended foreward far out of the proper line of the arch. The models made at the time, Figs. 1 and 2 clearly show the condition. The nose was also involved in the deformity, especially the septum, see Figs. 1 and 4. The fissure in the

Fig. 1.

lip, Figs. 1 and 3, was at the oral margin three-fourths of an inch broad, and at the nostril one-half inch. Through the hard palate the fissure was a little over one-quarter of an inch in width, enlarging to one-half an inch in the soft palate, Fig. 2. This made a continuous cavity from the tongue to the base of the skull. The vomer and turbinated bones were markedly abnormal, especially the vomer, which had an osseous process extending backward from its free margin to the posterior wall of the pharynx.

Under the direction of Dr. W. H. Dorrance, of the dental department, the first endeavor was made with a plate to draw together the margins of the maxillary bones. This plate was of rubber, with springs. All of the teeth and as much of the alveolar process as possible were embraced by the plate. Owing to the age of the patient, the teeth were so slightly erupted that they offered no retaining form and it was necessary to ligate the plate to the teeth so as to hold it firmly in place.

To make this plate, an accurate impression was taken of the teeth and process, on each side separately, as much as possible of the teeth and process was included. The point of contact of the plate with the lower teeth was built up so as to present an even surface for mastication. The location and size of the lugs necessary for the attachment of the wire springs were indicated on the wax moulds to be carved in the plaster after the investment had been made. These plates were made of hard rubber, and fitted to the mouth, then an impression was taken with them in position and the plates removed with the impression. After this impression had been poured a model was obtained with the plates in, in a secure and relative position for the adjustment of the springs. The springs, which were attached to lugs vulcanized to each side of the plate, were made of number 22 gauge piano wire, in the form of a right angle with two small coils at the angle. These were placed in the lugs on the buccal side of the plate, and a German silver wire passed around the front through the lugs made to hold it in position, being attached to the springs

Fig. 2.

on either side. A large U-shaped spring was then made for the posterior part of the plate of the same kind of wire, but number 18 in size. This spring was formed to fit close to the plate that it might not interfere with the tongue. A very slight amount of tension was put upon these springs at first, and the plate put in position in the mouth. This was worn constantly for about four weeks except as it was removed every day for cleansing and re-adjustment. The width of the fissure in the alveolar process was seven thirty-seconds of an inch. After wearing the plate for three weeks the edges of the process were in actual contact, the larger part of the change was made during the last five days.

Very careful attention was given to the regulation of the pressure to prevent undue irritation. After bringing the edges of the cleft together an operation was made to unite the palatal processes and to correct the curve of the arch. A fracture was made in the line of the right pre-maxillary suture and the pre-maxillary bones thus loosened were placed in their proper places, this brought the incisor teeth into the arch.

Fig. 3.

In Fig. 3 it will be seen that the left central incisor stood at an angle of about 50 degrees to its proper axis. This tooth was extracted with the intention of replacing it afterwards but there was no room for it. The contiguous surfaces were then freshened and secured with a silver ligature. To prevent displacement of the parts until a hard rubber splint could be made, the parts were invested in soft modelling compound over the surface of which Iodoform was sprinkled. The rubber splint was frequently perforated to facilitate cleansing, at the same time it was made strong enough to securely hold the parts in place until union had occurred.

For ten days after the operation the mouth was washed every two hours with per manganate of potassium and calendula to prevent infection.

The result of the operation was a perfect curve to the arch and a complete union of the anterior third of the maxilla and the alveolar process. An operation was then made on the lip with very satisfactory results, as seen in Figs. 4 and 6.

Fig. 4.

It was difficult to understand a word that the boy said when he first came to the college, but after this operation he was able to articulate with tolerable distinctness. No words in which the palatal muscles had a part could be articulated distinctly because of the defects in the soft and part of the hard palate, which were practically unchanged except that the cleft in the latter had been somewhat narrowed. It did not seem practicable to unite these by an operation, and accordingly it was decided to make an artificial substitute in the form of a plate and soft rubber velum.

A serious difficulty in the way of making this plate, was the age of the patient and the consequent retarded eruption of the teeth, making it difficult to secure anchorage for the plate. Support was had, however, partially by atmospheric pressure and partially by pressure on the process above the teeth, see Fig. 5, and by a band on the central incisor. This plate was made of rubber, in order that it might be easily changed on the approach of an incoming permanent tooth, or that it might be made over after the usual changes had taken place in the mouth. The plate supports one tooth as seen in Fig. 5, the space occupied by it will, however, be quite filled up by the natural teeth when the permanent denture is completely erupted.

A brief description of the method of producing the plate and velum is as follows:

An extension was soldered to a number 12 impression tray, to give support to the impression material throughout the extent of the hard and soft palate. The impression was taken with magnifique, and on the first introduction but little attention was directed to anything except to the proper distribution of the material. After the material had slightly chilled, it was removed, and the posterior part of the impression made quite soft in hot water, as was also the upper surface of the entire impression. It was then returned to the mouth and pressed firmly but carefully to place. This gave minute detail and the soft palate with a minimum of distortion. No effort was made at this time to get

Fig. 5.

an impression of the superior border of the palate about the cleft. A plaster model was made from this impression and upon it a special tray was made with which an accurate impression of the borders of the cleft could be secured. This tray consists of a piece of block tin rolled to the thickness of number 16 plate and cut heart shape, about an inch and a half in length by an inch in breadth. To this is soldered a piece of number 14 brass wire doubled to form a handle about five inches long; one end of the wire was allowed to pass up through the tin and curve backward, thus forming a loop to retain the magnifique in position. This tray with the magnifique in place, was passed well back into the fauces and carefully drawn forward into the cleft, and the patient instructed to swallow to cause the palatal muscles to force the borders of the soft palate and divided uvula into the material to their normal position. It is necessary to repeat this process several times so that the sensitive parts may become accustomed to the presence of the foreign material and allow the impression to be taken without gagging. In this case it was impossible to get an impression without securing local anæsthesia with cocaine. From this impression a model was made in two sections, and on this model was formed a composition test piece, to be used to make a hard rubber pattern, from which was made the soft rubber velum.

Fig. 6.

In this case the parts were so sensitive and the patient so young that it was thought best to make a velum somewhat smaller than would be required at a later time, until such time as he should have become accustomed to the appliance. Two vela were made differing somewhat in size and shape. For the first one, for which no duplicates would be required, the writer as an experiment pursued the following course: The test piece was made as it would be for forming a hard rubber pattern of very hard magnifique, and was very carefully shaped but no effort was made to get a bright finish. • Since the soft rubber cannot be polished, the pattern from which it is made must have a very high finish. This was accomplished by covering the entire surface of the carefully prepared composition pattern with a gloss procured in the following manner: Gum damar was dissolved in carbon bisulphide, making a perfectly clear fluid of about the consistency of glycerine; the pattern dipped in this and the surplus fluid hastily shaken off; it was then rotated and turned for a few moments to prevent the fluid from running while hardening, which it does very rapidly. This produced a finish similar to glass and much higher than is possible on the hard rubber. It was then invested so that the investment division in the flask came at the margin of the upper valve, consequently the soft rubber velum was entirely free from the ridges always produced by the divisions of the investment when the hard rubber or metal patterns are used, and which it is impossible to entirely remove.

If a hard rubber pattern of this piece had been desired, the mold for it could have been made in a few moments from the soft piece.

The pin hole in the test piece having been counter-sunk at either end its location was very distinct on the soft piece and the perforation was readily made with an Ainsworth rubber-dam punch.

The model upon which the plate was to be made, was then cut to allow the soft piece to go into place with the pin, by which it would be attached to the plate, in position. An impression was then taken in plaster of the lower valve of the velum, including the platinum attachment of the pin, the model having been first thoroughly soaked. The whole was then removed from the model, and the velum removed from the pin. Then by returning this impression to the model, the lower valve of the velum was reproduced in plaster with the pin invested in its exact position. On this model was then constructed the plate which should support the soft rubber velum seen in Fig. 5.

It will be seen from the model, Fig. 4, that there is a slight protrusion at the oral margin of the lip, opposite the point of union. This will in a few months entirely disappear, owing to the contractility of newly formed connective tissue. In Fig. 5 a space will be seen between the right permanent central and the right temporary cuspid. The permanent lateral is already in sight and will fill this space.

The models of the face, Figs. 1 and 4, are facsimiles of the conditions, reproduced from plaster impressions.

Although the proper relation of the parts had been restored by this appliance he had yet to learn to talk, for this only made it possible to form the tones properly. As a means for instructing him what the proper position was for the various tones, a number of models were made like Fig. 5, on which was indicated the spots which the tongue must touch to form the various tones.

Although at his age he cannot appreciate readily the methods of using the Obturator, and although it will take months for the heretofore unused muscles to become very active, he could when he had worn it less than a week, converse so distinctly that anyone could readily understand him. It was several days before he could remove and replace the obturator himself, because of the extreme sensitiveness of the parts. The degree to which the deformity is visible, can be judged by reference to Fig. 6.

## Figures and Tables

**Fig. 1. f1:**
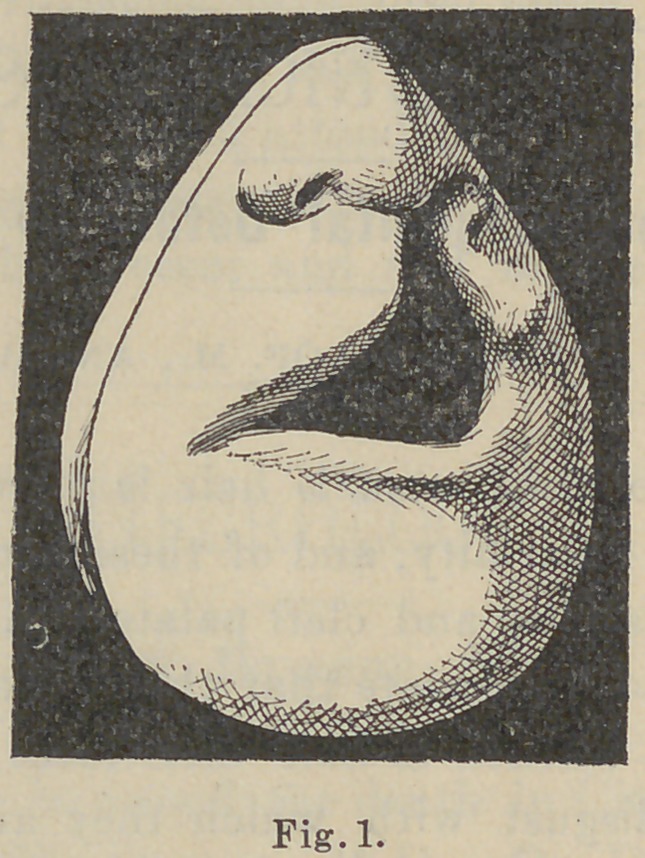


**Fig. 2. f2:**
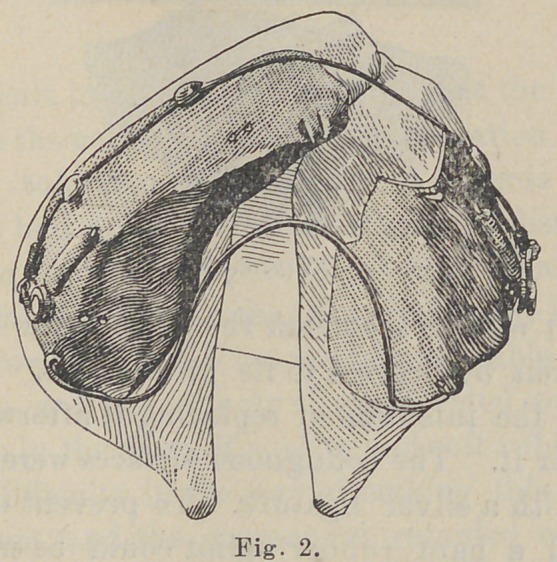


**Fig. 3. f3:**
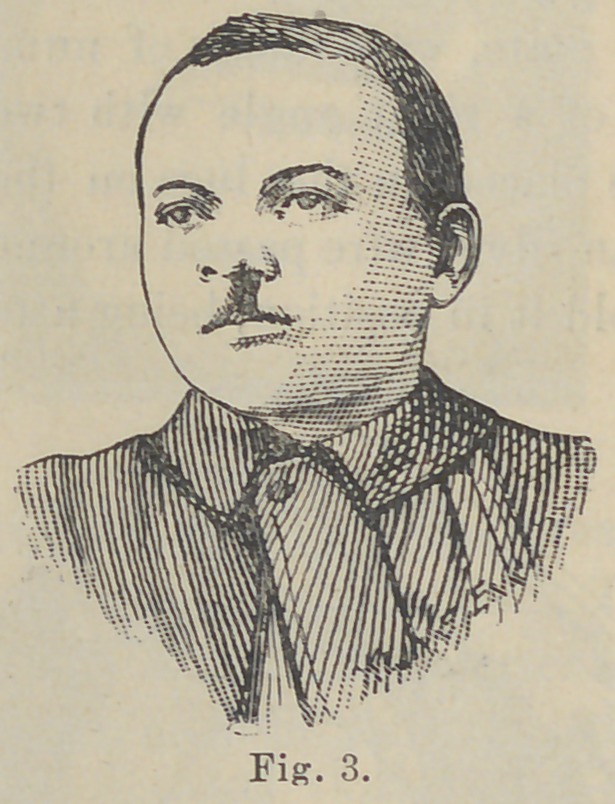


**Fig. 4. f4:**
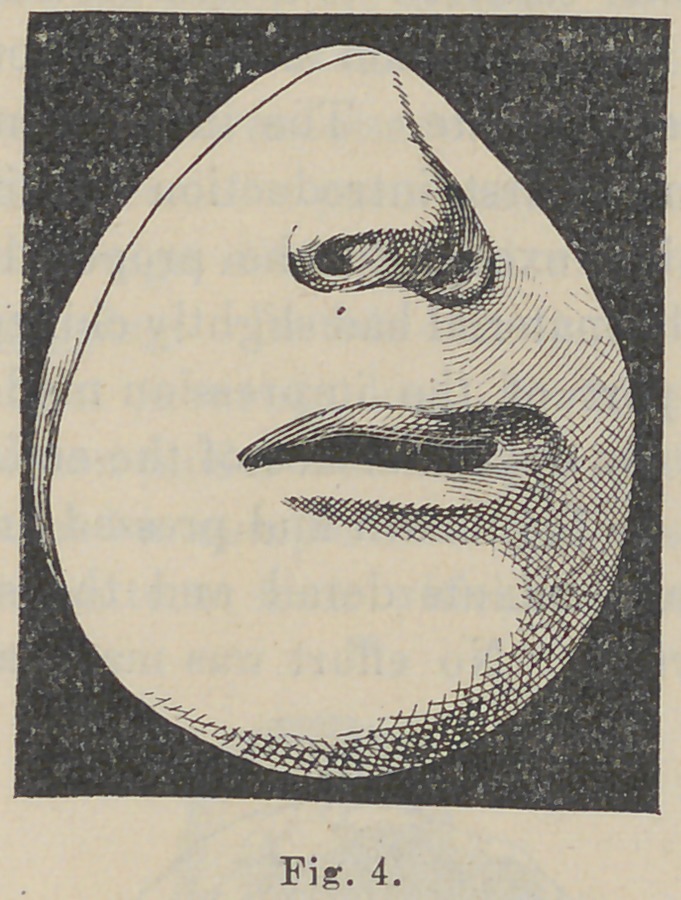


**Fig. 5. f5:**
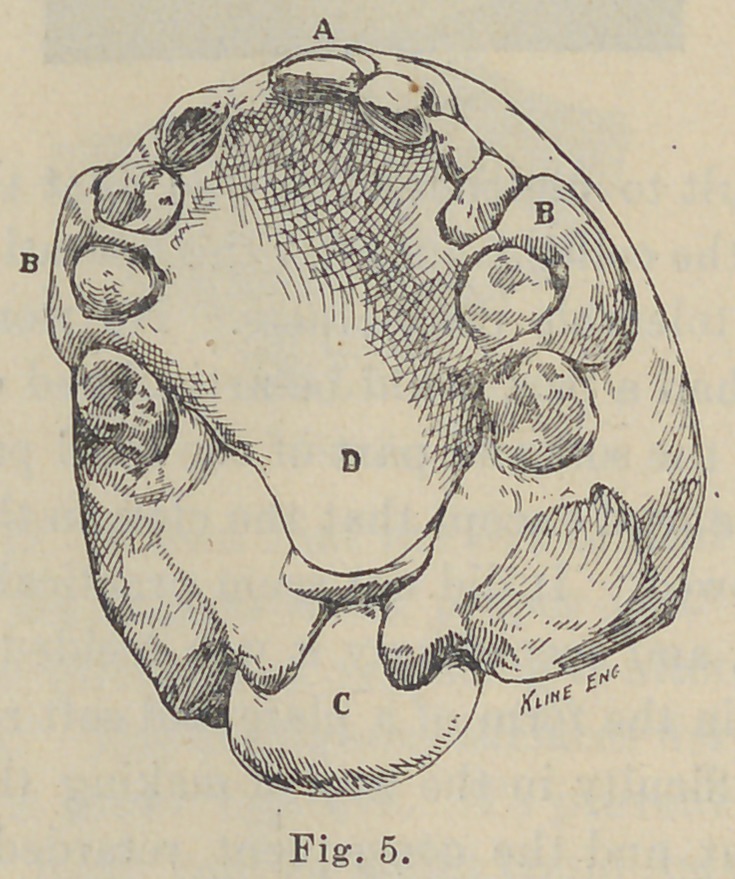


**Fig. 6. f6:**